# Effects of Integrating and Non-Integrating Reprogramming Methods on Copy Number Variation and Genomic Stability of Human Induced Pluripotent Stem Cells

**DOI:** 10.1371/journal.pone.0131128

**Published:** 2015-07-01

**Authors:** Xiangjin Kang, Qian Yu, Yuling Huang, Bing Song, Yaoyong Chen, Xingcheng Gao, Wenyin He, Xiaofang Sun, Yong Fan

**Affiliations:** Key Laboratory for Major Obstetric Diseases of Guangdong Province, Key Laboratory of Reproduction and Genetics of Guangdong Higher Education Institutes, The Third Affiliated Hospital of Guangzhou Medical University, Guangzhou, 510150, China; University of Kansas Medical Center, UNITED STATES

## Abstract

Human-induced pluripotent stem cells (iPSCs) are derived from differentiated somatic cells using defined factors and provide a renewable source of autologous cells for cell therapy. Many reprogramming methods have been employed to generate human iPSCs, including the use of integrating vectors and non-integrating vectors. Maintenance of the genomic integrity of iPSCs is highly desirable if the cells are to be used in clinical applications. Here, using the Affymetrix Cytoscan HD array, we investigated the genomic aberration profiles of 19 human cell lines: 5 embryonic stem cell (ESC) lines, 6 iPSC lines derived using integrating vectors (“integrating iPSC lines”), 6 iPSC lines derived using non-integrating vectors (“non-integrating iPSC lines”), and the 2 parental cell lines from which the iPSCs were derived. The genome-wide copy number variation (CNV), loss of heterozygosity (LOH) and mosaicism patterns of integrating and non-integrating iPSC lines were investigated. The maximum sizes of CNVs in the genomes of the integrating iPSC lines were 20 times higher than those of the non-integrating iPSC lines. Moreover, the total number of CNVs was much higher in integrating iPSC lines than in other cell lines. The average numbers of novel CNVs with a low degree of overlap with the DGV and of likely pathogenic CNVs with a high degree of overlap with the ISCA (International Symposium on Computer Architecture) database were highest in integrating iPSC lines. Different single nucleotide polymorphisms (SNP) calls revealed that, using the parental cell genotype as a reference, integrating iPSC lines displayed more single nucleotide variations and mosaicism than did non-integrating iPSC lines. This study describes the genome stability of human iPSCs generated using either a DNA-integrating or non-integrating reprogramming method, of the corresponding somatic cells, and of hESCs. Our results highlight the importance of using a high-resolution method to monitor genomic aberrations in iPSCs intended for clinical applications to avoid any negative effects of reprogramming or cell culture.

## Introduction

Human-induced pluripotent stem cellsare derived from differentiated somatic cells using defined factors. Like human embryonic stem cells, iPSCs are capable of unlimited proliferation and of differentiating into all cell types of the body. The generation of patient-specific iPSCs holds promise for regenerative medicine because they can provide a renewable source of autologous cells for cell therapy without the risk of immune rejection. Retrovirus- or lentivirus-based delivery systems have served as mainstream methods of generating iPSCs, although three independent groups successfully generated mouse iPSCs using tetraploid complementation to conclusively demonstrate that iPSCs are equivalent to ESCs in terms of pluripotency[1–3]. However, genomic integrations of reprogramming factors in virally generated iPSCs not only cause insertional mutagenesis but also lead to residual expression of reprogramming factors in iPSCs and their derivatives. Several recent studies reported that virally induced iPSCs harbor genetic or epigenetic and transcriptional abnormalities—including dysregulation of imprinted genes, CNVs, accumulation of point mutations, aberrant methylation patterns and other chromosomal aberrations—that are either pre-existing or generated during reprogramming[4–7]. Thus, iPSCs that maintain original genomic integrity and do not carry integrated viral vector sequences or transcription factor DNA are highly desirable for clinical applications.

Many reprogramming methods have been employed to generate human iPSCs without genome-integrating DNA elements. These methods use, for example, episomal vectors, adenoviral vectors, Sendai viral vectors, plasmids, synthetic mRNA, miRNA, protein transduction and small molecules[8–12]. All of these methods have disadvantages, such as low reprogramming efficiency, a requirement for serial transgene deliveries, or success being limited to only certain types of somatic cells, such as commonly used fibroblasts[13]. Of all the reagents used in these non-integrating reprogramming methods, episomal vectors are particularly appealing because they are easy to manipulate and because they allow a relatively high efficiency compared to the other non-integrating methods. Recently, Okita et al. modified their episomal vectors to simultaneously encode more than one reprogramming factor to generate human iPSCs efficiently[9]. Chou et al utilized an improved episomal vector and successfully generated iPSCs from blood cells[14]. Thus, the generation of human iPSCs based on improved episomal vectors is believed to be efficient, free of genomic integration of transgenes, and representative of a step forward in the development of autologous and allologous stem cell therapy.

Genomic stability is critical for clinical applications of human iPSCs. Because genetic aberrations have been strongly associated with cancer, it is important that iPSCs prepared for clinical use are free from cancer-associated genomic alterations. Recently, several studies have identified genomic abnormalities such as chromosomal aneuploidy and translocations, megabase-scale duplications and deletions, and point mutations in iPSCs. Laurent et al have reported increased subchromosomal CNVs in pluripotent cell samples, with the enriched CNVs located in specific genomic regions[5]. They also found increased numbers of deletions in human iPSCs associated with tumor-suppressor genes, whereas duplications of oncogenes are found in iPSCs that have been cultured for extended periods of time. Gore et al have reported that point mutations accumulate particularly in oncogenic pathways in otherwise karyotypically normal human iPSCs[15]. Hussein et al found that early-passage human iPSCs have more CNVs than intermediate-passage iPSCs or ESCs[7]. However, most of the iPSCs described in these studies have genomic abnormalities generated from integrating reprogramming methods. In this study, we performed high-resolution HD genotyping on 10 iPSC lines, the two matched fibroblast and AF lines from which they came, and four hESC lines. The iPSC lines were reprogrammed in our lab using an integrating method (four-factor lentiviral) and a non-integrating method (episomal vector). We found that the iPSC lines generated by non-integrating methods have lower incidences of genomic aberrations than those generated by integrating methods. Our results suggest that a high-resolution method such as HD genotyping should be strongly recommended for monitoring genomic aberrations throughout iPSC preparation to minimize any effects of acquired genomic aberrations in clinical applications.

## Materials and Methods

### Cell culture

All experiments were approved by the ethical committee of The Third Affiliated Hospital of Guangzhou Medical University. The tissue donors provided their written informed consent for participation. Briefly, the donors were informed that their tissues would be used for scientific research and that the research results would be published in a scientific research journal but that their names will not be listed in the paper. The donors were not compensated for their donation. Human amniotic fluid (AF) cell lines, fetal fibroblast (FF) cell lines and human ES cell lines were independently established in our laboratory [16,17]. AF cells were cultured in AmnioMAX-II Complete Medium (Invitrogen, Carlsbad, CA, USA). Fetal fibroblast (FF) cell lines were cultured in fibroblast medium (Dulbecco's Modified Eagle's medium (DMEM) supplemented with 10% fetal bovine serum (FBS) (Hyclone, Logan, UT, USA), 1 mM glutamine (Gibco), l% non-essential amino acids (NEAA) (Gibco), and 100 IU/ml penicillin/streptomycin (Gibco)). Cells were infected with STEMCCA lentiviral supernatants generated by the transfection of 293T packaging cells as previously described[18]. To generate non-integrating iPSCs, AF cells or FF cells were transfected with Episomal iPSC Reprogramming Vectors (Invitrogen, A14703) by electroporation or by transfection with the CytoTune-iPS 2.0 Sendai Reprogramming Kit (Life Technologies, A16518). Transfected cells were then plated onto vitronectin-coated culture dishes according to the manufacturer’s instructions.

Human ESC and iPSC lines were cultured on Matrigel-coated tissue culture dishes (ES qualified; BD Biosciences) with mTeSR1 (STEMCELL Technologies, Vancouver, BC, Canada) at 37°C and 5% CO2 in a 100% humidified atmosphere incubator. The culture medium was refreshed daily until the cells were ready to passage or harvest. Cells were passaged using 1 mg/ml dispase (Gibco) every 3–4 days.

### DNA purification

Genomic DNA was extracted and purified with QIAamp DNA Blood Mini Kit (QIAGEN, Germany) according to the manufacturer’s instructions. RNA digestion was performed at 37°C for 1 hour using RNase (QIAGEN). The concentration and quality of the DNA samples were determined using a spectrophotometer (Nanodrop 2000, Thermo Scientific, USA) and 1% agarose gel electrophoresis.

### CNV and SNP genotyping

The Affymetrix Cytoscan HD array (Affymetrix, Santa Clara, CA, USA), which interrogates 2,696,550 copy number markers across the human genome, was used to characterize cell lines of interest to determine whether there are different associations with de novo CNVs generated using different reprogramming methods. For each sample, 250 ng of input genomic DNA was amplified and labeled according to the manufacturer's instructions[19]. Hybridization of the labeled product using a GeneChip Hybridization Oven 645 (Affymetrix) was followed by washing using the GeneChip Fluidics Station 450 (Affymetrix) and scanning using the GeneChip Scanner 3000 7G (Affymetrix). Data processing was performed in Chromosome Analysis Suite 2.0 (CHAS2.0) software (Affymetrix), via the standard reference library provided by the manufacturer based on the Hidden Markov Model. The segments filter was set to 300 kb and 50 markers for CNV and to 3 Mb and 50 markers for LOH.

### Validation of CNV

qRT-PCR was performed to validate CNV calls. SYBR Green assays were performed in triplicate and the predicted copy number was calculated based on the equation: CN = 2*(2^(-ΔΔCt)).

### Accession numbers

The Gene Expression Omnibus (GEO) accession number for the Affymetrix Cytoscan HD array data which has been submitted to Gene Expression Omnibus in this paper is GSM 1704973–GSM 1704991.

## Results

**Fig 1:**
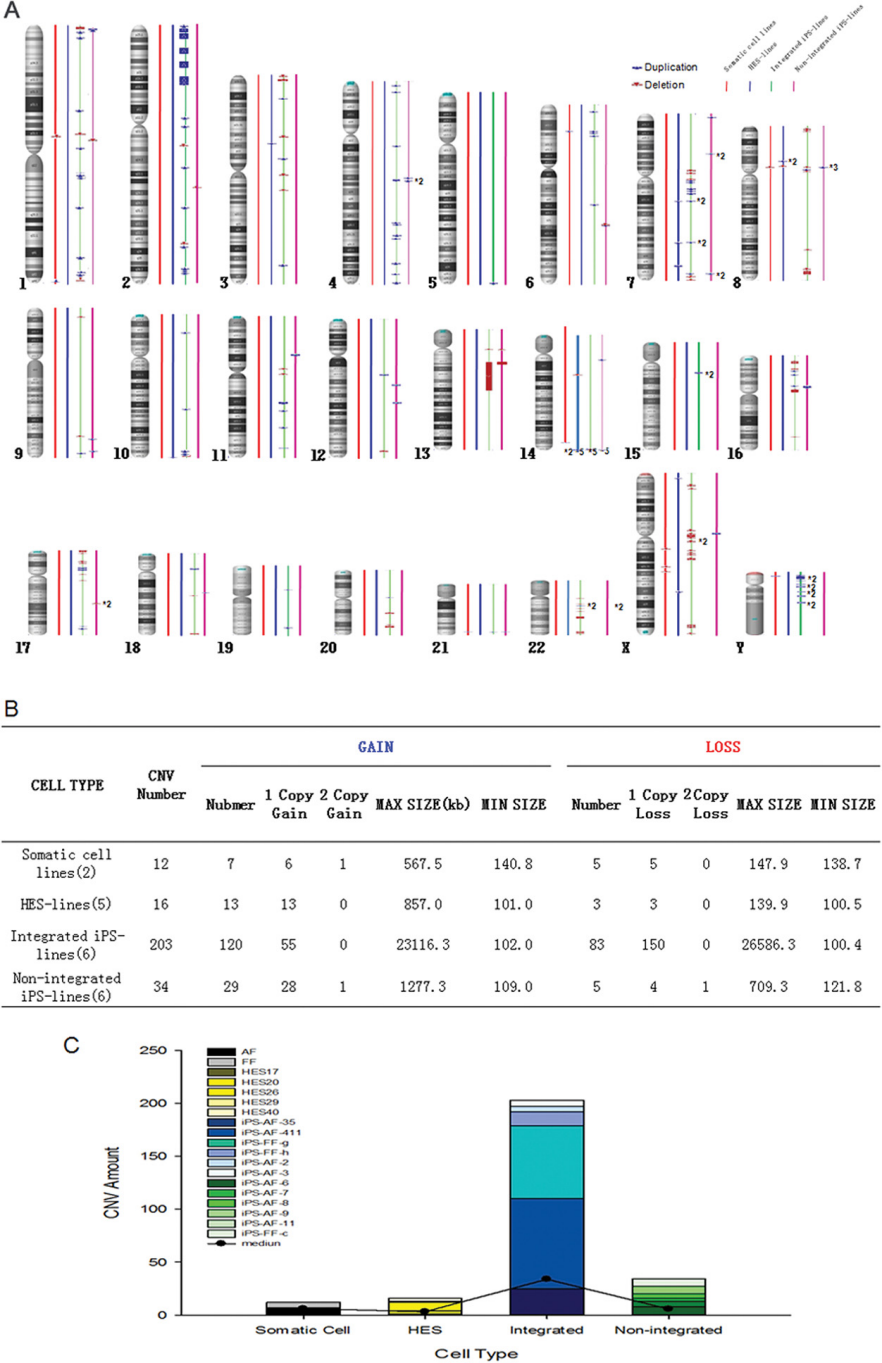
CNV analysis in somatic cells, hESCs and hiPSCs. A) Genomic representation demonstrating the sharp difference in the number of somatic cells, hESCs and hiPSCs. Blue boxes represent amplifications, and red boxes represent deletions, with sizes varying with CNV coverage. B) Comparison of total numbers and average sizes of CNVs between different cell lines. C) Detailed numbers of CNV in each line and the average value of particular cell types.

Previous studies have shown that CNVs are generated during the reprogramming process. To investigate the effect of integrating and non-integrating reprogramming methods on genomic integrity of iPSCs, high-resolution CNV and SNP genotyping (Affymetrix Cytoscan HD array) was performed on 19 samples comprising 2 somatic cell lines, 5 hESC lines, 6 iPSC lines generated by the integrating method and 6 iPSC lines generated by the non-integrating method (Table 1). These cells clearly showed hESCs characteristics (S1 Fig). Fig 1A shows a map of the areas of CNVs identified in all the samples.

**Table 1:** A list of human cells analyzed for a genomic integrity state in this study.

Cell ID	Cell Type	Reprogramming Method	Ability of Differentiation
AF	Human amniotic fluid cells(P3)	None	None
FF	Human fetal skin fibroblast cells(P5)	None	None
HES17	Embryonic Stem Cells(P29)	None	Pluripotent
HES20	Embryonic Stem Cells(P25)	None	Pluripotent
HES26	Embryonic Stem Cells(P25)	None	Pluripotent
HES29	Embryonic Stem Cells(P24)	None	Pluripotent
HES40	Embryonic Stem Cells(P26)	None	Pluripotent
iPS-AF-35	Induced Pluripotent Cells (P17)	Integrating	Pluripotent
iPS-AF-411	Induced Pluripotent Cells (P15)	Integrating	Pluripotent
iPS-FF-g	Induced Pluripotent Cells (P12)	Integrating	Pluripotent
iPS-FF-h	Induced Pluripotent Cells (P12)	Integrating	Pluripotent
iPS-AF-2	Induced Pluripotent Cells (P15)	Integrating	Pluripotent
iPS-AF-3	Induced Pluripotent Cells (P15)	Integrating	Pluripotent
iPS-AF-6	Induced Pluripotent Cells (P11)	Non- Integrating	Pluripotent
iPS-AF-7	Induced Pluripotent Cells (P10)	Non- Integrating	Pluripotent
iPS-AF-8	Induced Pluripotent Cells(P12)	Non- Integrating	Pluripotent
iPS-AF-9	Induced Pluripotent Cells (P11)	Non- Integrating	Pluripotent
iPS-AF-11	Induced Pluripotent Cells (P12)	Non- Integrating	Pluripotent
iPS-FF-c	Induced Pluripotent Cells (P15)	Non- Integrating	Pluripotent

Numbers in parenthesis with P indicate passage in culture on the cells used in the genomic integrity analysis.

### Comparison of CNVs in somatic cells, hESCs and iPSCs

**Fig 2:**
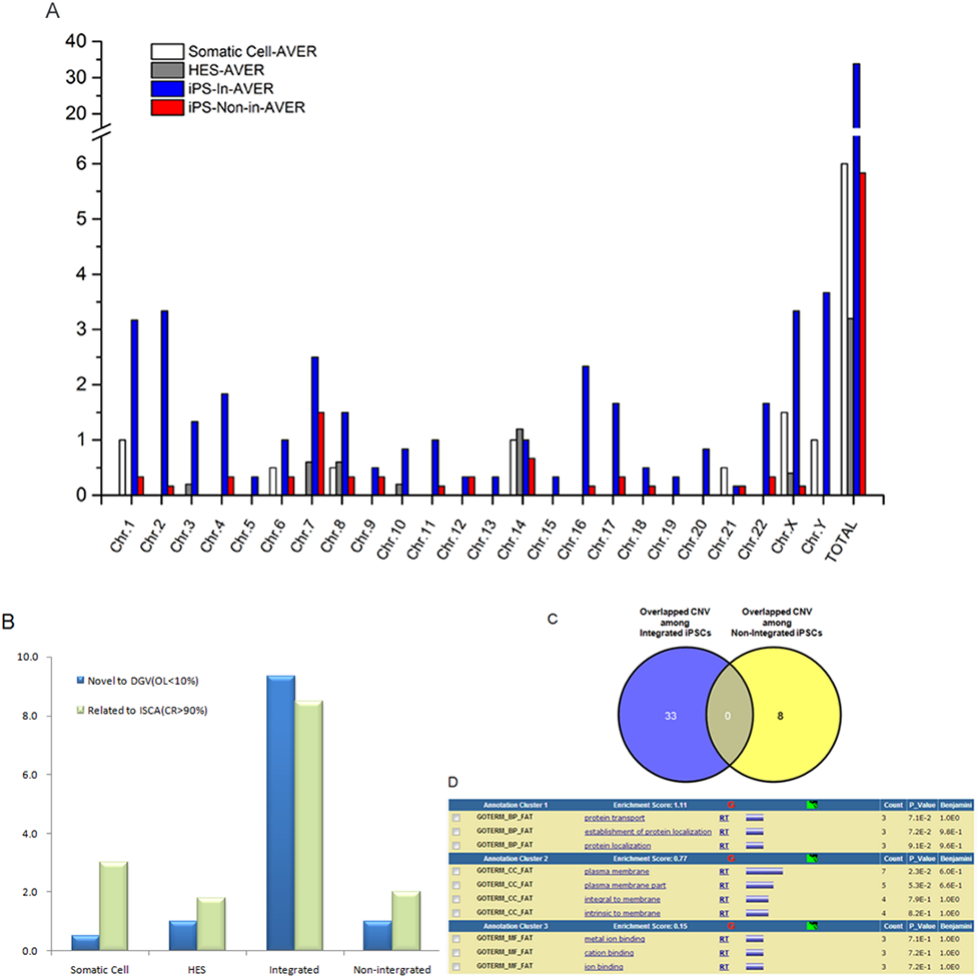
Comparison of CNVs induced by different reprogramming methods. A) Analysis of CNV distribution among chromosomes in somatic cells, hESCs and hiPSCs. B) Average number of novel CNVs with low degrees of overlap with DGVs (blue bar) and likely pathogenic CNVs with high degrees of overlap with the ISCA database (green bar) in somatic cells, hESCs and hiPSCs. C) Venn diagrams for total CNVs acquired after integrating or non-integrating reprogramming of hiPSCs. Total numbers of overlapping CNVs (n>2) in integrating (blue) and non-integrating (yellow) iPSC lines are displayed for representative hiPSC lines. The overlapping areas indicate the number of CNVs that are common between cell lines generated by the two methods. D) GO analysis of the overlapping CNVs among the integrating hiPSC lines.

Large deletions (>1 Mb) of chromosome 13 were observed in the integrating line iPS-FF-2. In lines iPS-FF-g and iPS-FF-h, 7 large deletions were identified in chromosomes 1, 3, 8, 10, 16, 17 and 22. The line iPS-FF-h had five large duplications of the short arm of chromosome 2. A large deletion of chromosome 13 was observed in iPS-AF-3 (Fig 1A). The maximum sizes of the duplication and deletion were 23116.3 kb and 26586.3 kb, respectively, and were both found in the integrating iPSC lines (Fig 1B). Moreover, the number of total CNVs was greater in integrating iPSC lines than in the other cell lines (Fig 1B). The average length of 3-copy duplications was significantly higher in the integrating iPSC lines (826.1 kb) than in the other lines. The median number of CNVs (>100 kb) in the non-integrating iPSC lines (5.7) was approximately the same as that in hESCs (3.2) and somatic cells (6.0) but six-fold lower than in the integrating iPSC lines (33.8) (Fig 1C). Despite the considerable variation in the number of CNVs among these samples, the average numbers of aberrations in the integrating iPSC lines were overall significantly higher than that in the somatic cells, hESCs and non-integrating iPSC lines (Fig 1B). Among the pluripotent stem cell lines, the genomic aberrations were evenly distributed throughout the genomes in iPSC lines. In contrast, the distribution of CNVs in the four hESCs samples was highly skewed; CNVs were detected only in chromosomes 3, 7, 8, 10, 14 and X (Fig 2A) in hESCs. These results suggest that there had been a positive selection in culture for iPSCs with large aberrations that greatly changed their genomic stability from that of their parental cell lines (Fig 1C).

In addition to these large deletions and duplications, recurrent CNVs associated with each reprogramming method were individually identified in the human pluripotent stem cell samples. We had previously observed that the validation rate for small deletions, which are usually detected at a higher frequency in pluripotent cells, was significantly lower than that for small duplications. Therefore, for subsequent analyses, we focused on recurrent de novo duplications relative to the parental cell line. In both the lines iPS-FF-g and iPS-FF-h, we saw duplications of approximately 100 kb located at 7q21.11 (this duplications contain an OMIM gene: SEAM3E) as well as 7q31.32 (this duplications contain an OMIM gene: CADPS2) (Fig 2A). These duplications were found neither in the parental somatic cell samples nor in the non-integrating iPSCs, implying they might be specifically induced by the integrating reprogramming method.

### Catalogue of the identified CNVs in diverse cell lines

On the other hand, no significant differences in the total number of CNVs were observed in the pluripotent cells derived from the two somatic cell sources (Fig 2A). No aberration was recurrently located on a particular chromosome in any somatic cell-derived iPSC line.

We generated a list of 33,506 common CNVs identified in 1,038 healthy individuals and of DGVs. These common CNVs could be considered to be mostly functionally neutral. We designated as “novel CNVs” the set of CNVs that were identified in our study and overlapped to a low extent (<10%) with common CNVs. The average number of novel CNVs was 0.5 in somatic cells, 1.0 in human ESCs, 1.0 in non-integrating iPSCs, and 9.3 in integrating iPSCs. The novel CNV fraction was significantly higher in early-passage integrated iPSC lines, while the fraction in non-integrating iPSC lines was as low as the fraction in somatic cell lines (Fig 2B). A set of CNVs from our study that had high coverage rates in the ISCA database (>90%) were designated as ISCA-related CNVs, the average number of which in integrating iPSCs was high. The average number of ISCA-related CNVs were 3.0, 1.8 and 2.0, respectively in somatic cells, hESCs and non-integrating iPSCs, respectively, and was 8.5 in integrating iPSCs. The novel CNV fraction was significantly higher in early-passage integrating iPSCs (Fig 2B).

In AF cell lines and AF-derived iPSCs, 33 CNVs were detected that overlapped with CNVs in integrating iPSCs, while eight other CNVs were detected that only overlapped with CNVs in non-integrating iPSCs (Fig 2C). Gene ontology (GO) analysis revealed that genes containing CNVs particularly induced by the integrating reprogramming method were related mainly to protein transport, plasma membrane and ion binding (Fig 2D).

### LOH and SNP in human iPSCs

**Fig 3:**
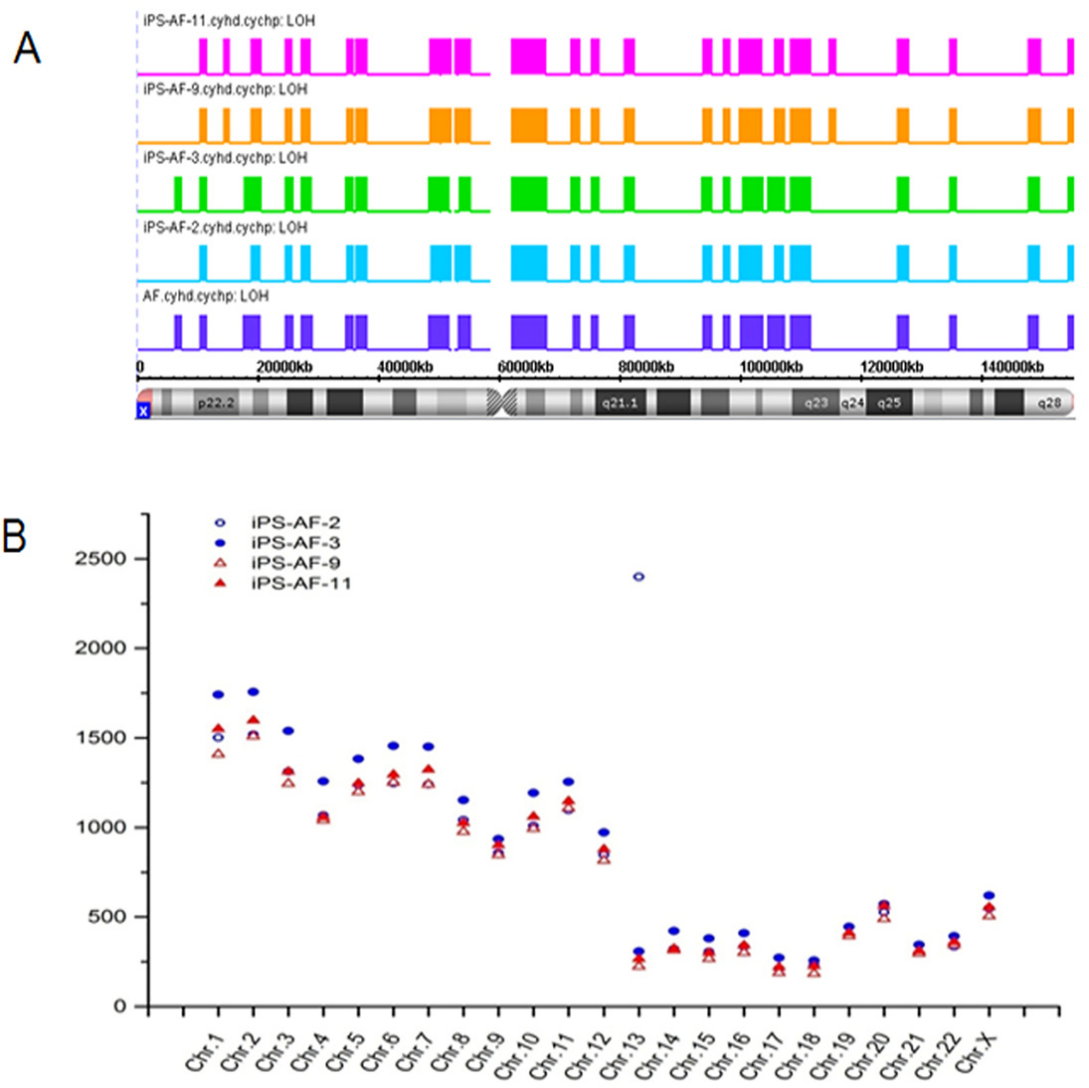
Analysis of LOH and SNVs in the genomes of integrating hiPSCs and non-integrating hiPSCs derived from the same parental cell line. A) LOH changes in the X chromosome caused by the reprogramming method. B) Analysis of SNV distribution among chromosomes.

In both integrating and non-integrating iPSC lines, 72.5% of 2-copy LOH fragments (>1500 kb, >50 marker count) originated from the parental cells and were stably maintained without any recovery of heterozygosity. LOH fragments were recurrently found in the X chromosome, which accounted for 25.4% of the total number (Fig 3A). The integrating line iPS-AF-3 had the exact same LOH pattern as that of its parental cells. Both the lines iPS-AF-9 and iPS-AF-11 had induced loss of heterozygosity in Xp22.2 and Xq23 relative to the AF cells. Because LOH was calculated based on the call for the SNP marker, these data support the hypothesis that a significant number of mutations occur during or shortly after reprogramming and then were selected during the expansion of iPSCs. Further analysis based on SNP markers revealed that, compared to the parental cells, integrating iPSC lines displayed more different SNP calls than did non-integrating iPSC lines (Fig 3B).

### Genetic mosaicism in human iPSCs

**Fig 4:**
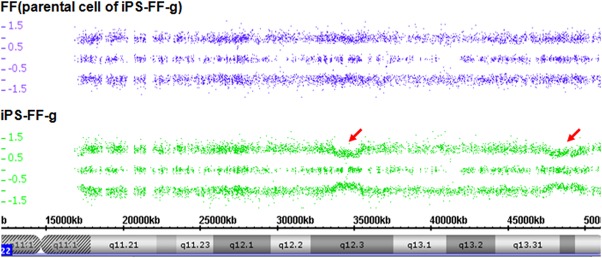
Chas data browser representation of the mosaicism of chromosomes in integrating hiPSCs compared to its parental cells.

Genetic mosaicism resulted from the de novo generation of CNVs, followed by selection for less damaged cells during propagation. From the microarray data, no mosaicism was detected in non-integrating iPSC lines, while mosaicism was clearly evident in approximately 38%-48% of integrating iPSC lines (Fig 4). Moreover, in the tested cell lines, mosaicism was occasionally found in human AF cells but not in hESCs or human fibroblasts.

## Discussion

iPSCs present an ideal system for distinguishing between the effects of reprogramming and those of cell passaging on genome stability[7,20]. This study is unique in combining non-pluripotent and pluripotent samples to detect cell type-specific as well as reprogramming method-specific genomic aberrations in a high-resolution microarray platform that enables the detection of kilobase-length aberrations, which are not detectable by karyotyping or low-resolution microarrays. We mainly aimed to evaluate the effects of iPSCs generated by integrating and non-integrating reprogramming methods on genomic stability. Four hESC lines were used as controls, although some of the abnormalities might have been present in the embryos from which the cells were derived. The parental cells from which iPSCs were derived were also analyzed, because the difference between the pluripotent cells and cultured somatic cells cannot be ignored.

Schlaeger et al performed a small-scale genome-wide aCGH analysis and found that the majority of CNVs were preexisting and that the frequency of possible de novo aberrations was uniformly low among representative patient-derived human iPSC lines obtained by diverse methods[13]. In this study, we intended to identify method-specific differences in CNV loads via microarray analysis with a high probe density. The results presented here indicate that the integrating iPSC lines contain numerous genomic aberrations. Interestingly, non-integrating iPSCs and hESCs were found to contain more duplications, while integrating iPSCs were found to contain more deletions, than somatic non-pluripotent cells. These findings further substantiate the persistence of the effects of these two reprogramming methods of inducing pluripotent cells as well as of the subsequent selection forces[13]. According to our results, early-passage non-integrating iPSCs generated by the episomal or Sendai-virus (SeV) method were pluripotent and had fewer genomic anomalies compared to those generated by the virus method, which suggests that the non-integrating method should be considered more reliable, resulting in fewer genomic rearrangements in iPSC lines[13]. Episomal and SeV methods are the most commonly practiced reprogramming strategies to date[21,22]. These methods are based on plasmid DNA that can be easily generated according to cGMP-compatible processes[23]. Because SeV reprogramming is efficient with a low workload, increasing method-adoption rates have been reported for SeV[13]. Though it has been reported that SeV viral sequences are completely absent in most lines at higher passages, it is worth investigating in detail its dynamic effects on genomic stability.

Hussein et al. concluded that the reprogramming process is associated with a high mutation rate in the resultant early-passage human iPSC lines: novel CNVs in human iPSCs were mostly generated during the reprogramming process[7]. Early-passage cells then endured strong selection pressure and lost the majority of their de novo mutations[5]. Although our study was based mainly on a microarray approach, the high density and coverage of SNP and CNV probes enabled us to identify copy number changes, site mutations, and genetic mosaicism in tested cell lines. These mutations might confer a growth or survival disadvantage to the cells and therefore be selected against, eventually resulting in a CNV profile similar to those found in hESCs. Given that early-passage iPSCs were collected to focus on the influence of the reprogramming procedure, some genetic changes appear to result generally from selection pressures in the continuous cell culture, rather than specifically from the reprogramming. We extended the analysis by asking whether all the CNVs observed in iPSCs in this study pre-existed in the parental somatic populations at low frequencies or were introduced during reprogramming and the subsequent culturing. We compared the loads of CNVs and the mosaicism in the parental cell lines from which all tested iPSCs were derived and found more copy number variants (>50 kb) and mosaicism (>10%) in AF cells than in fibroblast cells. However, as regards iPSCs derived from these two types of cells, no significant differences in either CNV load or mosaicism were detected in this study. This observation suggests that aberrations in iPSCs are induced mainly by reprogramming and are not selected from pre-existing aberrations in somatic cells during the process.

Differential expression and DNA methylation in somatic cells, hESCs and iPSCs have already been reported in previous studies[6,17,24]. All reprogramming methods resulted in similar expression and DNA methylation patterns, with a small portion of genes showing method-specific expression[25]. A similar observation was made from a DNA methylation analysis that showed that only 0.14% of CpG sites were differentially methylated in episomally derived iPSC lines compared with virally derived ones[17]. These results show that diverse reprogramming strategies have major differences in their effects on genome structure but not on gene regulation.

The possible mechanisms responsible for reprogramming-induced CNVs are still under exploration. A series of recent studies investigated mutations reminiscent of those induced by replication stress and found CNVs located in regions of genomic fragility, such as common fragile sites and subtelomeric regions[4,26]. Unlike the results of other previous studies, no recurrent regions of duplication on chromosomes 12 and 20, which lie in close proximity to known pluripotency genes, were observed in our study. One explanation could be that the iPSC samples were of an early passage, although low passage number does not in itself ensure genetic integrity. A higher level of novel CNVs were detected in integrating iPSCs, suggesting the presence of a greater replication stress in those lines. Meanwhile, previous studies showed that the level of reactive oxygen species increased during reprogramming, which might contribute to the incidence of mutations in the genome[27]. The gene expression profiles of iPSC lines from both integrating and non-integrating methods will be further investigated to identify any differences in enzymatic activities relevant to ROS.

Genomic stability is critical for the clinical use of induced pluripotent stem cells and should be assessed frequently at the endpoint of every treatment. The results of our present study highlight the importance of determining the molecular mechanisms involved in reprogramming somatic cells to be pluripotent, as well as the necessity for optimizing reprogramming and culture conditions to improve genetic stability of iPSCs and their safety for clinical cell therapy.

## Supporting Information

S1 Fig Immunohistochemistry of the stem cell-specific surface antigens Oct4, Nanog and Tra-1-60 in AF-iPSCs and FF-iPSCs and teratoma formation of those iPSCs by subcutaneous implantation into NOD/SCID mice. The iPSCs differentiated into various tissues, including ectoderm (neural tissues), mesoderm (cartilage) and endoderm (glandular tissues).
(TIF)
