# Supplementary figures and images for: Effects of Integrating and Non-Integrating Reprogramming Methods on Copy Number Variation and Genomic Stability of Human Induced Pluripotent Stem Cells
Source: PLoS One. 2015 Jul 1;10(7):e0131128. doi: 10.1371/journal.pone.0131128 (PMC4488894; doi:10.1371/journal.pone.0131128)

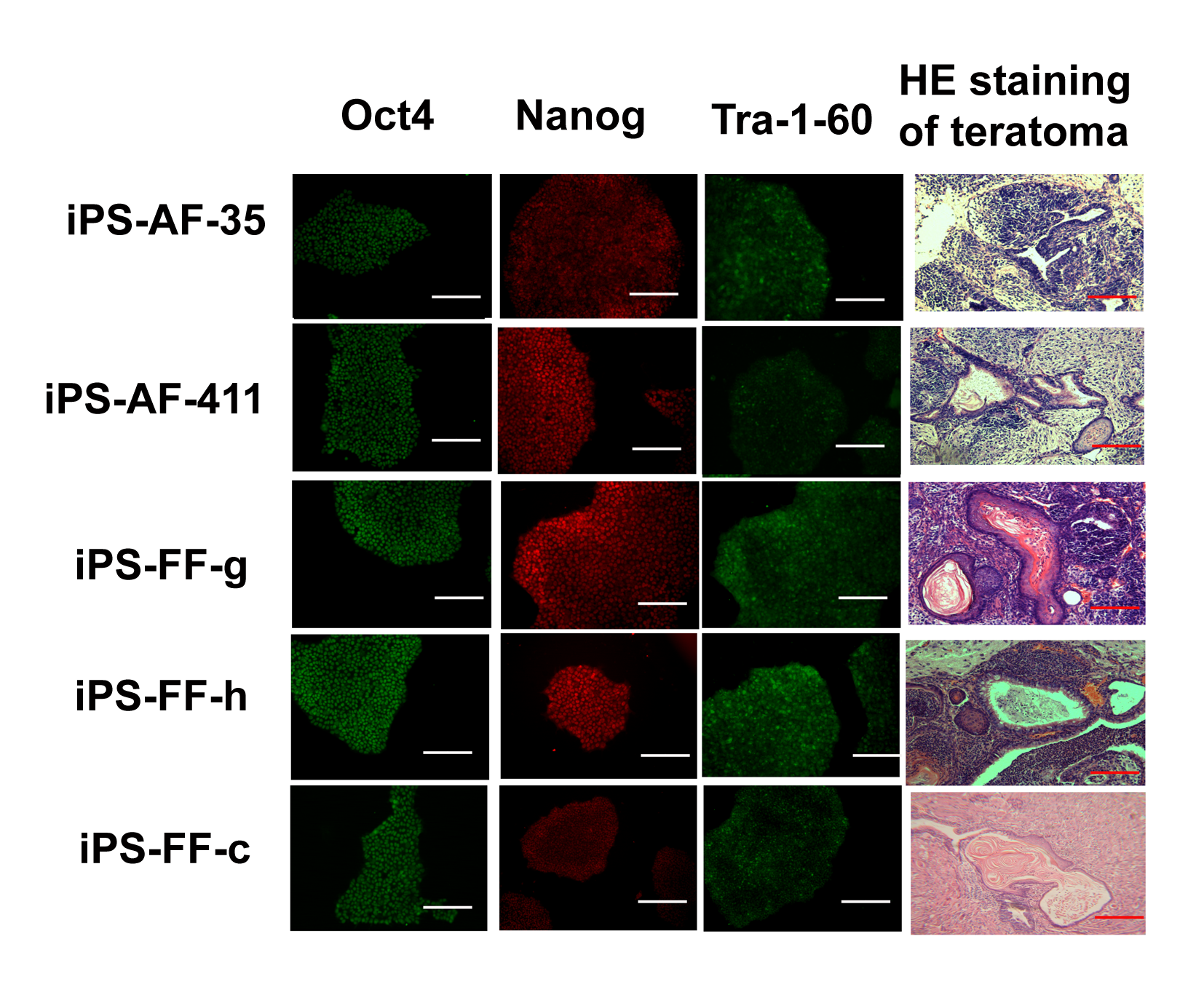

Supplement: S1 Fig — The iPSCs differentiated into various tissues, including ectoderm (neural tissues), mesoderm (cartilage) and endoderm (glandular tissues). (TIF) [file pone.0131128.s001.tif]
